# Improving Refill Adherence in Medicare Patients With Tailored and Interactive Mobile Text Messaging: Pilot Study

**DOI:** 10.2196/mhealth.8930

**Published:** 2018-01-30

**Authors:** Rena Brar Prayaga, Erwin W Jeong, Erin Feger, Harmony K Noble, Magdalen Kmiec, Ram S Prayaga

**Affiliations:** ^1^ mPulse Mobile, Inc Encino, CA United States; ^2^ Medicare Medication Therapy Management Kaiser Permanente Southern California Downey, CA United States; ^3^ Regional Pharmacy Clinical Operations Kaiser Permanente Southern California Downey, CA United States

**Keywords:** patient activation, patient engagement, medication adherence, refill management, text messaging, interactive, NLP, Medicare, disease management, technology acceptability model

## Abstract

**Background:**

Nonadherence is a major concern in the management of chronic conditions such as hypertension, cardiovascular disease, and diabetes where patients may discontinue or interrupt their medication for a variety of reasons. Text message reminders have been used to improve adherence. However, few programs or studies have explored the benefits of text messaging with older populations and at scale. In this paper, we present a program design using tailored and interactive text messaging to improve refill rates of partially adherent or nonadherent Medicare members of a large integrated health plan.

**Objective:**

The aim of this 3-month program was to gain an understanding of whether tailored interactive text message dialogues could be used to improve medication refills in Medicare patients with one or more chronic diseases.

**Methods:**

We used the mPulse Mobile interactive text messaging solution with partially adherent and nonadherent Medicare patients (ie, over age 65 years or younger with disabilities) of Kaiser Permanente Southern California (KP), a large integrated health plan, and compared refill rates of the text messaging group (n=12,272) to a group of partially adherent or nonadherent Medicare patients at KP who did not receive text messages (nontext messaging group, n=76,068). Both groups were exposed to other forms of refill and adherence outreach including phone calls, secure emails, and robo-calls from December 2016 to February 2017.

**Results:**

The text messaging group and nontext messaging group were compared using an independent samples t test to test difference in group average of refill rates. There was a significant difference in medication refill rates between the 2 groups, with a 14.07 percentage points higher refill rate in the text messaging group (*P*<.001).

**Conclusions:**

The results showed a strong benefit of using this text messaging solution to improve medication refill rates among Medicare patients. These findings also support using interactive text messaging as a cost-effective, convenient, and user-friendly solution for patient engagement. Program outcomes and insights can be used to enhance the design of future text-based solutions to improve health outcomes and promote adherence and long-term behavior change.

## Introduction

### Overview

Patient nonadherence affects 50% to 60% of chronically ill patients, and the cost of medication-related hospitalizations is $100 billion annually [1-3]. It is also associated with poor outcomes and progression of disease causing approximately 125,000 deaths and at least 10% of hospitalizations every year [4]. Seniors take an average of 7 medications per day, representing the highest number of prescribed medications for any age group [5].

Nonadherence is a major concern in the management of chronic conditions such as hypertension, cardiovascular disease, and diabetes where patients may discontinue or interrupt their medication for a variety of reasons. Patients are considered adherent when they take their medications (dose, time, frequency) as prescribed by their health care provider and as agreed to by the patient. Medicare populations adherence rates are often measured by pharmacy refill rates. The Centers for Medicare and Medicaid Services (CMS) uses the proportion of days covered (PDC), developed by Pharmacy Quality Alliance, to calculate adherence. Based on this, a patient who has a PDC rate of at least 80% is considered to be adherent.

Adherence is a particularly difficult problem among Medicare populations, and adherence rate is a key metric used by CMS to measure quality of a managed care plan. Approximately 32% of Medicare Part D patients are nonadherent to their diabetes, hypertension, and cholesterol medications [6]. Reasons for nonadherence may include side effects of the drug, cost of the drug, lack of perceived benefit, and/or forgetfulness.

### Use of Mobile Technology for Adherence

Studies and surveys are finding that digital health is not reaching most seniors, especially where there are socioeconomic disparities [7]. Among seniors who are identified as tech-savvy, 70% of those surveyed believe it’s important to be able to request prescription refills electronically, but fewer than half (46%) say they can do so today [8]. On researching mobile phone device ownership among seniors, we learned that while 78% of Americans aged 65 years and older own a mobile phone, only 34% own a smartphone [9,10]. We estimated smartphone ownership to be even lower among Medicare populations aged 65 years and older.

Text messaging using SMS (short message service) is ubiquitous, highly accessible, affordable, and commonly used across all income levels. It is also an effective channel for continuing to engage individuals in their health care once they leave the doctor’s office. Interactive text dialogues provide an opportunity for patients and health plan members to tap into health care resources and get support for healthy behaviors and long-term behavior change. Several studies have found that text messaging may serve as a scalable and effective means to improve medication adherence in chronic disease populations [11,12]. While there has been an interest in developing health technologies such as reminder applications [13-16] or automated phone reminders for older populations [17], a review of the literature reveals that very few programs have explored using text messaging with seniors to improve medication refill adherence [18,19].

We determined at the outset that since the target users for the program were an older and/or disabled population on Medicare, it would be important to focus on usability (ie, ease of use) and simplicity (ie, design for basic feature mobile phone instead of smartphone). We used Davis’ technology acceptance model (TAM) [20] as a guide to predict and optimize user acceptance of our solution as a viable and dynamic channel for interactive communication [21]. Therefore, our content strategy focused on usefulness and ease of use by providing simple instructions for authentication and task completion [22].

### Objectives

The program objectives were to assess the impact of an interactive and easy-to-use text messaging solution on medication refills and pharmacy operations and efficiencies. The target population consisted of partially adherent and nonadherent Medicare patients of a large integrated health plan (Kaiser Permanente Southern California, or KP) with 1 or more chronic diseases.

Our hypothesis was that patients receiving text message refill reminders (text messaging group) in addition to existing outreach would have a higher medication refill rate compared to the group that did not receive text messages (nontext messaging group).

## Methods

### Participants

The program began on December 7, 2016. All patients were Medicare members of KP with 1 or more chronic conditions (diabetes, hypertension, and/or high cholesterol). Patients in this program would be refilling 1 or more of the following 3 classes of drugs: oral diabetes medications, blood pressure medications (renin-angiotensin system antagonists), and statins.

There were approximately 5000 to 14,000 patients each week on the list who required pharmacy follow-up. These patients were pulled from 3 separate KP lists: (1) New Start: patients who filled their medication the first time in the calendar year and had a day supply remaining (DSR) of 0 to 30 days, (2) Near Goal: patients whose DSR ranged from –7 to 7 days and PDC ranged from 78% to 85%, and (3) Nonadherent: patients who had 2 fills within the calendar year and need to refill their medication by a specific date (Nonadherent date) in order to have a chance to improve their PDC to 80% or higher. The Nonadherent list patients were messaged in month 1 (December 2016) only.

Patients were divided into 2 groups:

1. Text message group (12,272/88,340, 13.89%): those who had opted in to receive text messages (as recorded within the health system’s electronic medical records [EMR]) and were on the weekly list for pharmacy follow-up (1000 to 4000 patients per week). These patients received text messages reminding them to refill their prescriptions. This group consisted of 12,272 patients who had opted in to receive text messages and did in fact receive text messages over the course of the program. Table 1 provides age and race/ethnicity breakdowns for this group.
2. Nontext message group (76,068/88,340, 86.11%): those who had not opted in to receive text messages or there was no indication of an opt-in (as recorded within the health system’s EMR) and were on the weekly list for pharmacy follow-up (4000 to 10,000 patients per week). This group consisted of 76,068 patients who did not receive text messages over the course of the program.

The text messaging group was one-fifth the size of the nontext messaging group because we were targeting only those Medicare patients who had opted in to receive text messages from KP. Both groups also received usual care which included phone calls and/or robo-calls reminding them to refill their prescriptions.

The Kaiser Permanente Southern California Institutional Review Board determined that this program did not involve human subject research and review was not necessary.

### Procedure

#### Solution Overview

**Figure 1:**
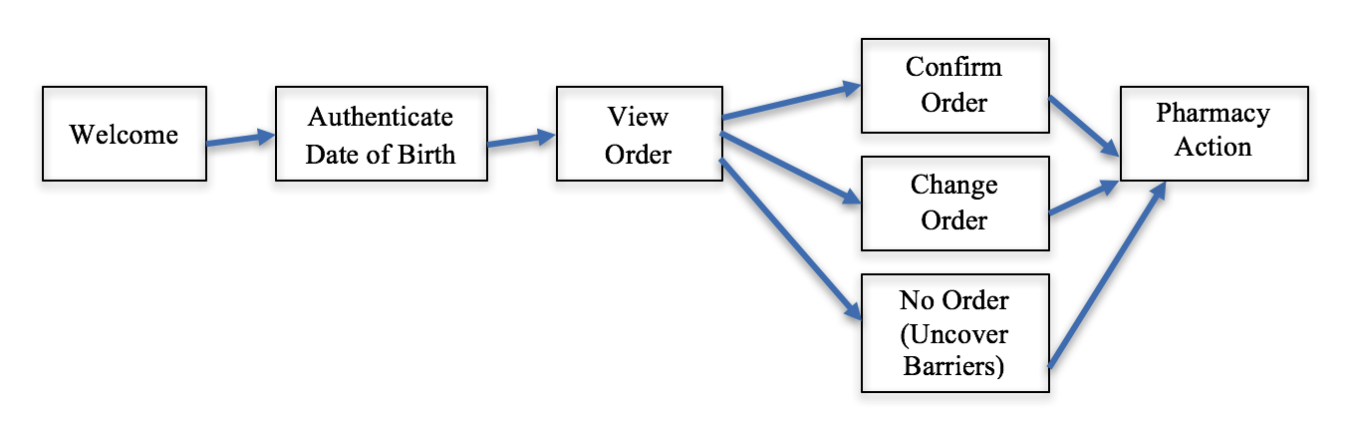
Overview of message flow within refill dialogue.

The mPulse Mobile platform delivers text messages to patients and members on behalf of health care companies. The platform consists of several components that together enable companies to interactively engage with their end-users about appointments, refills, gaps in care, or other health-related topics. Patients in the text messaging group received a refill reminder dialogue that consisted of a series of messages. All messages were written at a 6th grade readability level. The first message was a greeting reminding them that they were due for a refill. They were then prompted to enter their date of birth to authenticate and view their refill order (Figure 1).

Upon confirmation of the order by the patient, the KP pharmacy received a notification, and a KP pharmacist would process the refill and use the mPulse Engagement Console to inform the patient when it would be ready for pickup. Patients who did not respond to the initial message in the dialogue would receive follow-up reminders 2 hours later and again 24 hours later. They would then be moved through the same process (authentication, confirming refill order, etc). After confirmation of the order, there was no further communication with the patient. However, a small subset of patients was messaged again in a later dialogue because they failed to refill again the following month. A more detailed view of the dialogues and the process is provided in Multimedia Appendix 1.

**Table 1:** Characteristics of text messaging group.

Characteristic		Value, %
**Age, years**		
	Under 65	13.2
	65-70	39.7
	70-75	24.1
	75-85	18.9
	Over 85	4.1
**Race/ethnicity**		
	White	41.6
	Hispanic/Latino	30.0
	Black/African American	14.7
	Asian/native Hawaiian	10.9
	Unknown	2.75

**Figure 2:**
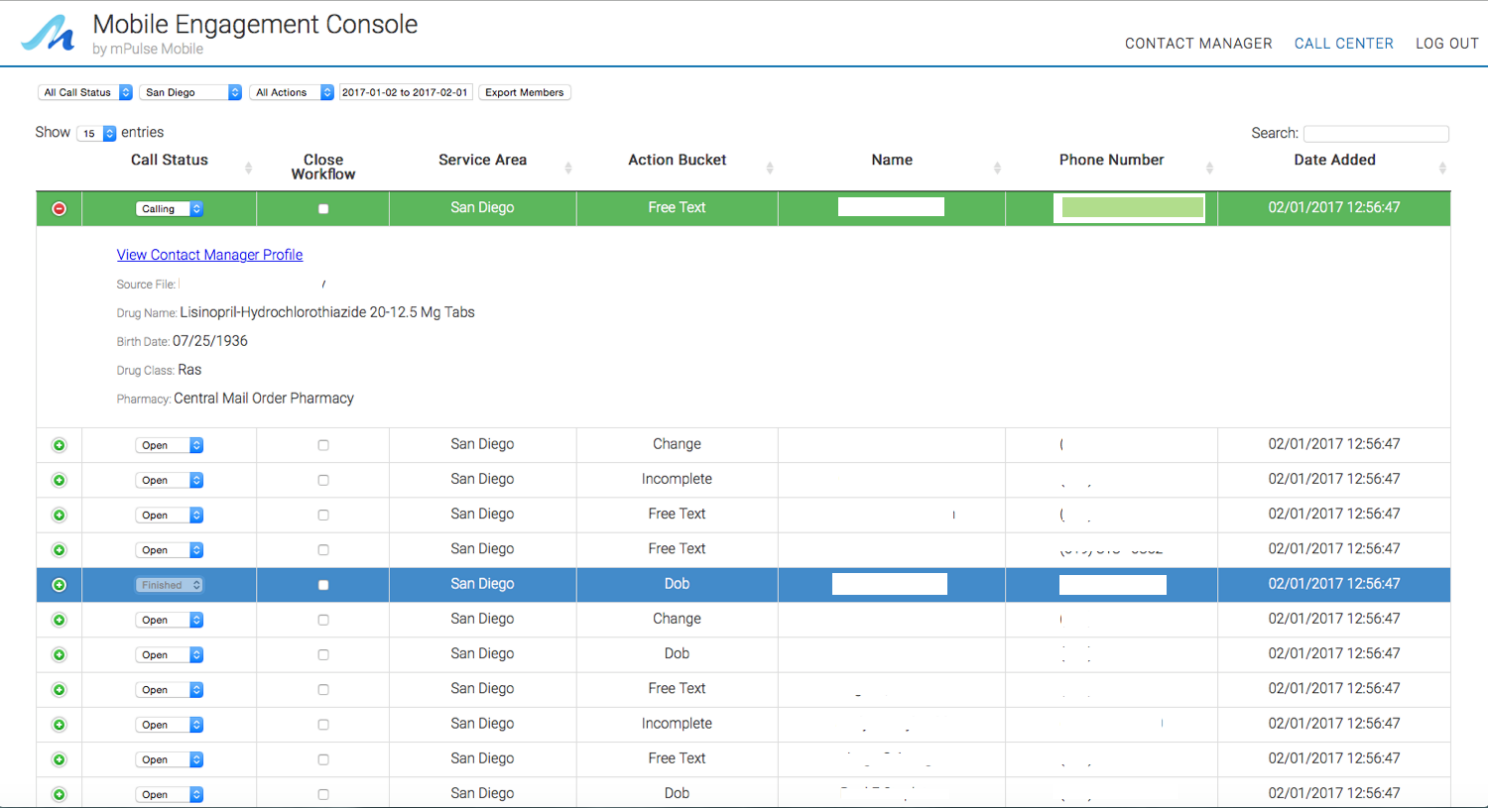
Engagement Console used to process refill requests and address other concerns via text.

Patients could move through the dialogue and authenticate their date of birth, complete a refill, ask for help, share reasons why they had not refilled already, or choose to opt out by using numeric or textual responses on their phone. The simplicity of the process allowed older users, who might also be more likely to have mobile phones instead of smartphones, to express their preferences and complete the process very easily.

If patients responded that they were experiencing side effects; did not believe the medication was helping them; wanted to change their medication, dose, or pharmacy; or had other concerns that might require follow-up, mPulse Mobile sent a daily list of members with pending questions or issues to the KP pharmacy for follow-up.

#### Dialogue Initiation

Refill dialogues were initiated at 10 am every Wednesday and Thursday to allow for a reasonable time frame within which patients could respond. Patients who texted STOP or 7867 (easier option for those without smartphones) would be opted out from the campaign and would not receive any further messages. Dialogues included tailored information to customize the message content (eg, name, date of birth, drug, pharmacy).

Patient information such as phone number, drug names, gender, name, mobile opt-in, level of adherence, and date of birth was used in 2 ways: to tailor message content for patients and initiate reminder dialogues to patients based on exclusion and combination logic. This logic helped avoid duplication and over-messaging (eg, member on multiple lists or multiple drugs would still receive a single dialogue). Patient information was provided weekly from the integrated health system and was used to perform dialogue assignments every week.

#### Refill Requests and Processing

Refill requests, questions, and concerns were handled by the pharmacy staff with a total of 8 staff members being trained on how to use the Engagement Console. To process refill requests or other concerns, staff would log on to a personalized view of the Engagement Console (based on their assigned medical center) and would be able to process any refill requests and other follow-up actions by initiating text messages directly to patients. They were provided with a list each week containing action buckets such as “refill requests,” “change requests,” “date of birth authentication failed or incomplete,” “help requests,” “concerns about side effects,” and “other free text responses” and prioritized their handling of these action items. Figure 2 provides a view of the Engagement Console. Additional images of the Engagement Console are provided in Multimedia Appendix 1.

Initially, processing refill requests via the Engagement Console took an average of 10 to 15 minutes. After the first week, time needed to process refill requests via the Engagement Console dropped to about 5 to 10 minutes per patient.

## Results

### Refill Request Rate

Our primary process measure was the number of refill requests. Of 13,195 dialogues initiated, we received a total of 2405 text messages requesting refills (Table 2). These requests were then processed by the pharmacy team and tracked separately.

Table 3 shows the number of patients targeted and the percentage who refilled by patient list. The refill request rate was highest for the Near Goal patients (1581/8206, 19.27%).

**Table 2:** Refill request rate for text message group by month.

Month	Refill dialogues, n	Refill requests, n	Refill request rate, %
Month 1	6776	1140	16.82
Month 2	3190	647	20.28
Month 3	3229	618	19.14
3-month total	13,195	2405	18.23

**Table 3:** Refill request rate for text message group by adherence level.

Adherence level	Refill dialogues, n	Refill requests, n	Refill request rate, %
Near Goal	8206	1592	19.40
New Start	748	120	16.04
Nonadherent	4241	693	16.34
Group total	13,195	2405	18.23

**Figure 3:**
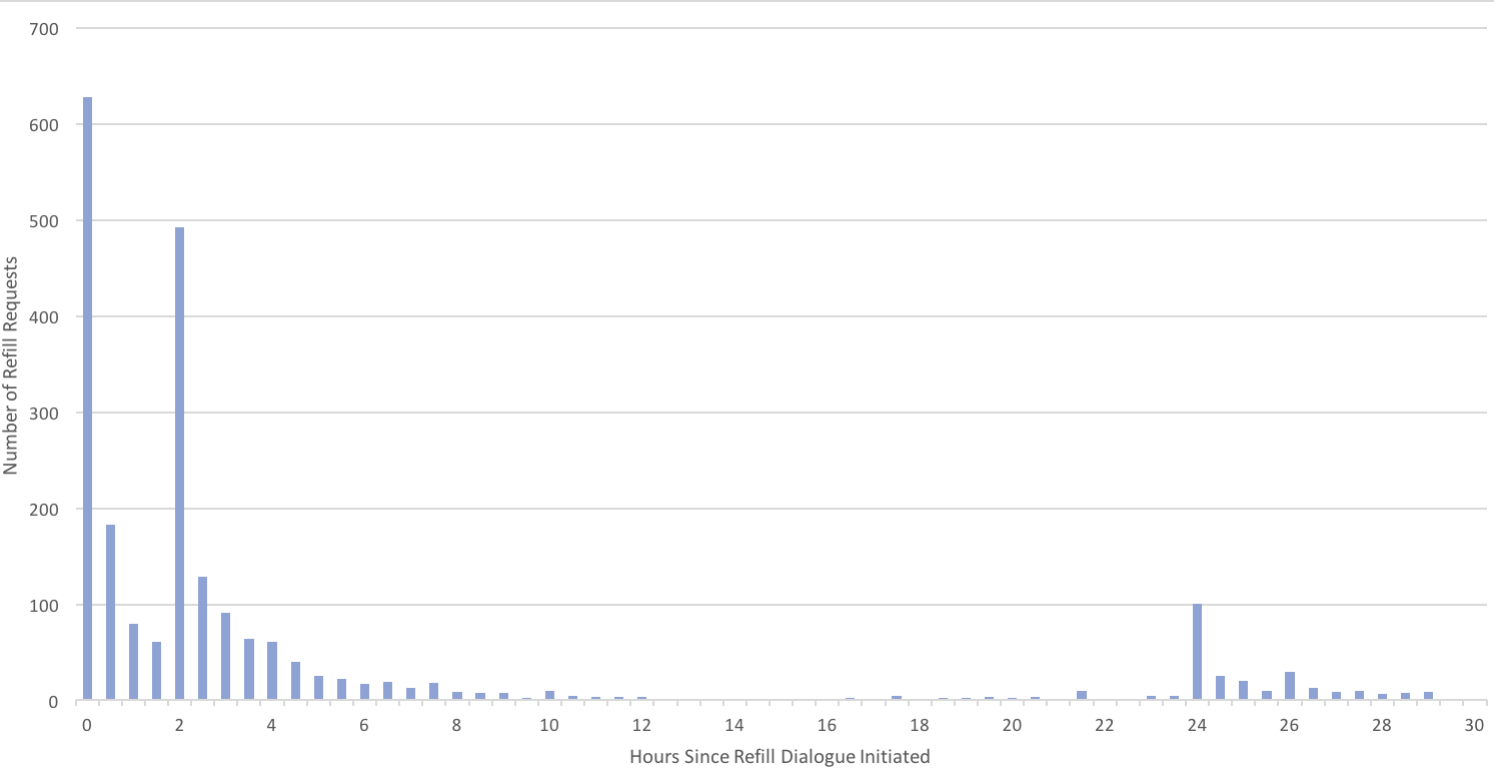
Refills requests by hour from initial reminder.

### Time to Request Refill

Of those who requested a refill, 37.33% (898/2405) did so within 2 hours of receiving the initial reminder, an additional 48.61% (1169/2405) refilled within 24 hours (after also receiving the 2-hour reminder), and the remaining 14.05% (338/2405) refilled after receiving the 24-hour reminder. As displayed in Figure 3, there are spikes in refill activity immediately after the initial message (0), after the 2-hour reminder (2), and the 24-hour reminder (24). On average, members engaged within 24 minutes of getting the first message, and the median time to move through the refill process after engaging was less than 2 minutes.

**Figure 4:**
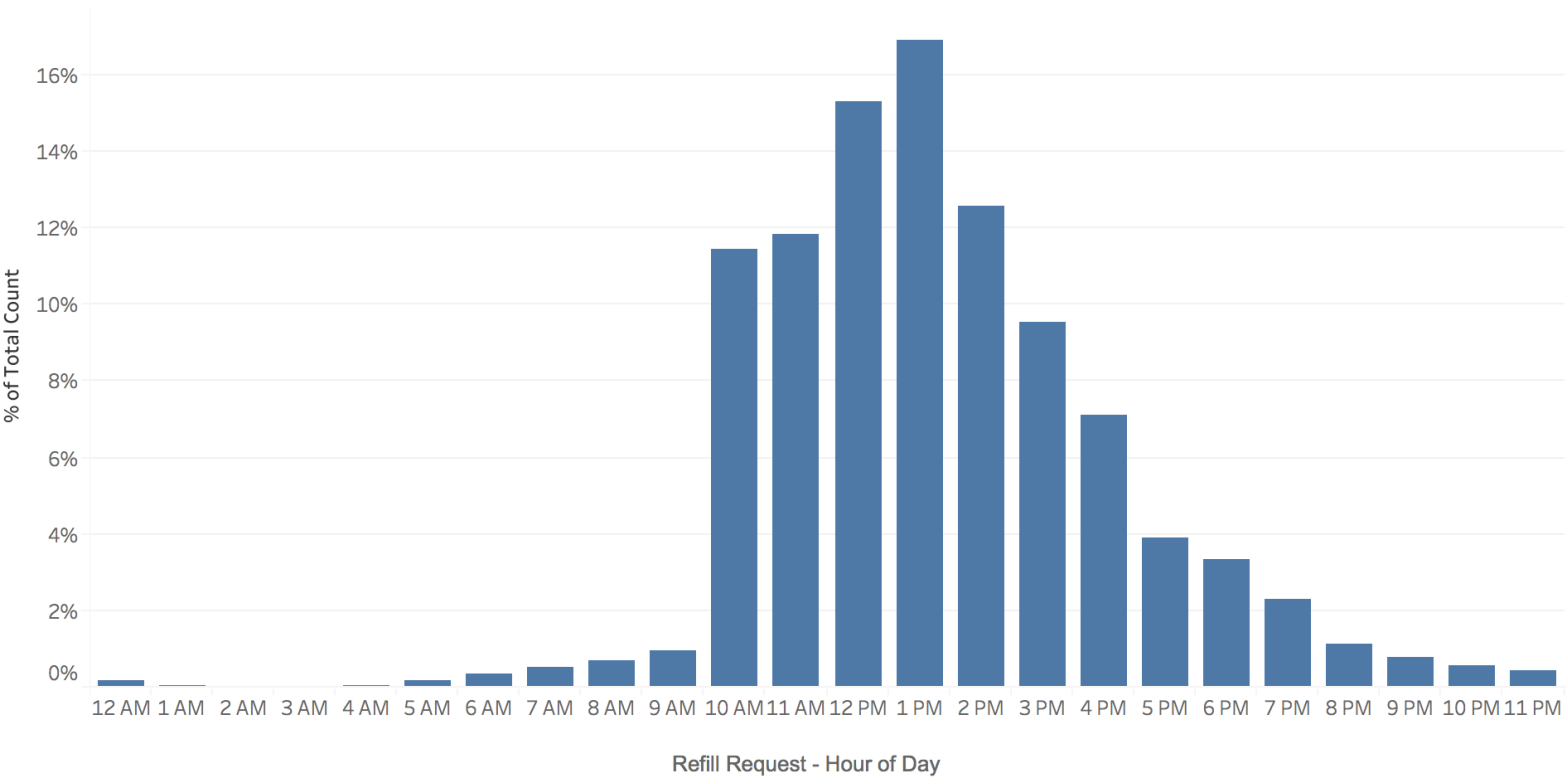
Percentage of refills requests by time of day.

Refill reminder dialogues were initiated between 10 am and noon on Wednesdays and Thursdays to allow for a reasonable time frame within which patients could respond. The bulk of refill requests (2210/2405, 91.89%) were made between the hours of 10 am and 6 pm (Figure 4). A majority of responses were received within the first 4 hours, and 81.12% (1951/2405) of responses were received within the first 8 hours.

We tracked refill request processing by pharmacy staff (total of 8 KP staff members) and noted that they collectively processed about 40 to 50 refills in an hour by the end of the first month of the program. Anecdotal feedback from KP pharmacy staff suggests that this improvement in processing refill requests has allowed them to double monthly refills.

### Refills Processed

Our primary outcome measure was the number of refills that could be attributed to the text messaging. We were measuring the incremental effect of text messages (in addition to usual care) in increasing medication refills.

**Table 4:** Differences in refill rates between the text message and nontext message groups.

Month	Text message group refill rate, %	Nontext message group refill rate, %	Difference in refill rates Percentage points	*P* value
Month 1	35.73	23.49	12.24	.001
Month 2	52.55	39.10	13.45	.001
Month 3	54.05	43.23	10.82	.001
3-month total	44.08	30.01	14.07	.001

In the text message group, 12,272 patients received refill reminders via text (in addition to other outreach) over the 3-month program, and 5410 (44.08%) of these patients refilled their medication. The nontext message group of 76,068 patients received flyers and other outreach but no text reminders, and 22,826 (30.01%) of these patients completed medication refills (Table 4). The text message group refill rates were much higher than the nontext message group rates, and the 14.07 percentage point difference in refill rates between the 2 groups was statistically significant (*P*<.001).

### Opt-Out Rates

The opt-out rate can be calculated in multiple ways and ranges from 1.02% to 5.09% depending on the calculation used. A total of 505 patients opted out over the course of the 3-month program. We have provided 3 different calculations in Table 5.

Here are the 3 different methods for calculating opt-out rates and rationale for each:

- Message level: This opt-out metric is calculated by dividing the number of members who opted out by the number of messages all members received. This measure helps us understand how long a member has stayed based on total volume of messages.
- Dialogue level: This opt-out metric is calculated by dividing the number of members who opted out by the number of dialogues all members received. This looks at the entire engagement in order to understand how well members received the program.
- Member level: This is the most common opt-out metric and is simply defined by dividing the number of members who opted out by the number of members at the beginning of the program. While this metric is useful, it does not factor in either program length or message volume and therefore presents a more coarse-grained view of member engagement and program value.

**Table 5:** Opt-out rates.

Approach for calculating opt-outs	Basis, n	Opt-out rate, %
Message level, messages	49,590	1.02
Dialogue level, dialogues	13,195	3.83
Member level, patients	9920	5.09

**Figure 5:**
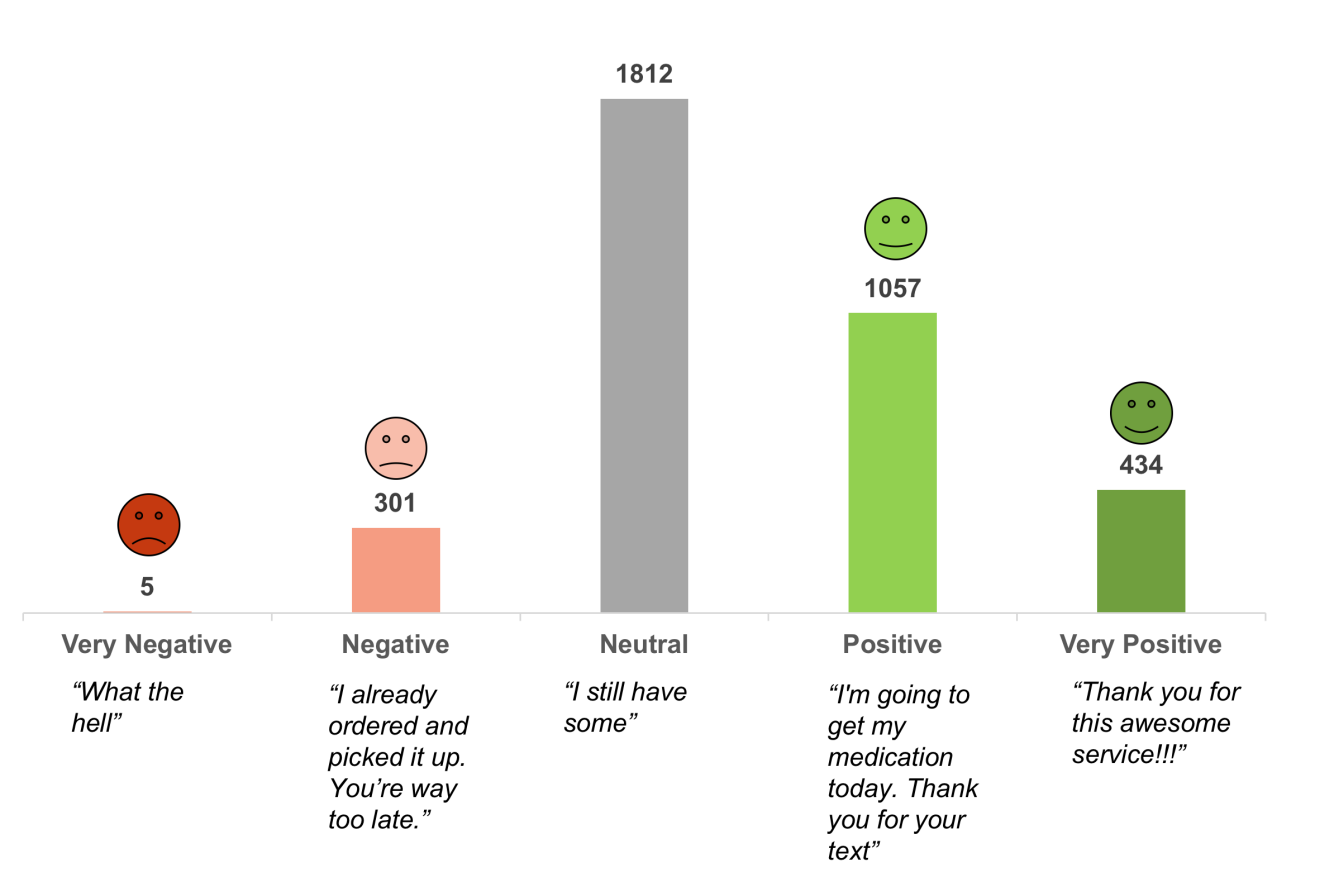
Sentiment in patient responses.

### Measuring User Experience

We analyzed patient free text responses to understand their experience and be more responsive. To do this, we used natural language processing to extract polarity, valence, and sentiment (very positive, positive, neutral, negative, very negative). For example, “Leave me alone” has a very different emotional tone than “Thanks so much for the reminder!” As shown in Figure 5, the largest subgroup of responses was neutral (1812/3609, 50.21%), followed by positive (1057/3609, 29.28%), very positive (434/3609, 12.03%), negative (301/3609, 8.34%), and very negative (5/3609, 0.14%).

### Ease of Use Survey Results

Another way in which we captured user experience was by asking patients directly. Starting in month 2, when patients completed a refill request, they received a confirmation message and were asked “Was this refill process easy to use?”

This question was intended to measure whether the TAM model’s “ease of use” consideration had been successfully embedded in the refill dialogue solution. In designing for usability, we had prioritized the importance of creating a text-based refill dialogue that was easy to use, easy to learn, did not cause users to generate many errors, and was helpful to users. Over 70.02% (890/1271) of those who were presented with the survey question completed it. Of the 890 unique patients who completed the survey, 850 (95.51%) responded “Yes,” and 40 (4.49%) responded “No.”

## Discussion

### Principal Findings

We studied the value of an interactive text message refill solution with a chronically ill and partially adherent or nonadherent Medicare population and observed a difference of 14.07 percentage points in refill rates between the text message group and comparison group (*P*<.001).

It is worth noting that patients in the texting group engaged at a much higher rate than predicted. We had estimated that the patient response rate would be between 10% and 20%, including stop requests, help requests, date of birth authentication attempts (successful and failed), refill requests, change requests, reasons for not refilling, and other free text responses. Our target refill request rate was 5% to 7% since we were messaging an older patient population. At the same time, we hoped that the ease of use of the refill dialogue might draw in more patients and nudge them toward completing their refill requests.

The program results far exceeded our expectations. Throughout the 3-month program, the response rate was around 37%, and the 3-month average refill request rate was 18%. We had also expected that since this was an older patient population the response time span might be stretched out a little longer, but this was not the case with over 80% of refill requests received within 8 hours of the initial reminder.

We used rules and basic natural language processing to improve recognition and handling of member responses over the course of the program, cut down unprocessed free text responses from 26% to under 16%, and reduced manual handling by pharmacy staff.

Overall patient feedback was very favorable and sentiment analysis of the responses revealed that patients were 5 times more likely to express positive sentiment than negative sentiment. Finally, almost 96% of the patients who completed refills via text message indicated that the solution was easy to use, and this strongly validated the TAM model and usability considerations that guided our design of the refill dialogues.

Although a cost-effectiveness analysis was not performed, interactive text messaging is inexpensive compared to manual calls or robo-calls. Finally, the high response rates and highly positive sentiment indicates improved patient engagement with their health care provider.

### Future Considerations

Our program incorporated basic demographic and psychographic data but did not tailor workflows based on the social determinants of health (ie, the conditions where people live, learn, work, and play and how these conditions affect their health risks and outcomes). This is an approach we plan to expand and implement in future programs. For example, how does living in a remote or rural area with no transportation impact refill behavior? How is income associated with refill rates? What about language and cultural barriers? This was a racially and ethnically diverse patient population. While the 3-month program used only English dialogues, the next phase would explore whether Spanish-speaking patients should be targeted differently and should also consider cultural and language barriers. We would also like to tailor content based on health literacy levels.

In future programs, we hope to combine demographic data (zip code, gender, age) with psychographic measures (adherence levels, past refill behavior, barriers to adherence, self-efficacy, stage of change, health beliefs) to develop a deeper understanding of the population being targeted to uncover health disparities and drive positive and sustained behavior change.

As we expand the program to other Kaiser Permanente regions, we expect to rely more heavily on machine learning–based natural language processing to improve recognition accuracy. Our machine learning–based natural language processing classifies a member’s response into most commonly occurring categories which, in turn, triggers appropriate actions. We use a model that is topic-specific and trained on data that is based on a combination of responses received within the program and gathered through other sources. While we also rely on human intelligence to validate and handle outliers and unexpected responses, our goal is to reduce manual processing of member queries and responses to less than 5% in future programs.

### Conclusion

Findings suggest that partially adherent or nonadherent Medicare patients who receive interactive text message refill reminders have significantly higher medication refill rates compared to similar patients who do not receive text message refill reminders. The program results demonstrate that this incremental value of interactive text messages increased refill rates by 14.07 percentage points in Medicare patients.

Results of the program include increased refill rates and high levels of patient engagement. These results should provide insights for developing similar models that represent an elevated standard of care within patient management.

## Appendix

Multimedia Appendix 1 Automated dialogues.

## Abbreviations

CMS: Centers for Medicare and Medicaid Services
DSR: day supply remaining
EMR: electronic medical records
KP: Kaiser Permanente Southern California
PDC: proportion of days covered
SMS: short message service
TAM: technology acceptance model
