# Supplementary material for: Improving Refill Adherence in Medicare Patients With Tailored and Interactive Mobile Text Messaging: Pilot Study
Source: JMIR Mhealth Uhealth. 2018 Jan 30;6(1):e30. doi: 10.2196/mhealth.8930 (PMC5811648; doi:10.2196/mhealth.8930)
Supplement: Multimedia Appendix 1 [file mhealth_v6i1e30_app1.pdf]

Multimedia Appendix 1: Automated Dialogues

A key component of the mPulse Mobile platform is the Engagement Engine which uses configurable automated dialogues for a specific purpose. An automated dialogue is an interactive exchange between the system and member with the intention of completing a task or process for the member. Automated dialogues can vary in purpose: refill a medication, cancel an appointment or get a health tip. The system supports these automated dialogues by using context within each interaction.

As part of the automated dialogue, responses received from a member or patient may not exactly match what was requested. For example, the message might request the member to enter “1” to view their order, but patients frequently respond in other ways – “#1”, “1.” “Option 1” or “Reply 1.”. Therefore, the automated dialogue embeds rules to handle these types of variations to continue the conversation in a meaningful and user-centered manner. Further enhancements include natural language processing to process responses. Such rules were used throughout the dialogue to accommodate for patient behavior. The program implemented 34 rules based on regular expressions (regex) that look for keywords and other patterns within the text. These rules match member responses and trigger a set of defined actions. Once a member response has been successfully handled, the automated dialogue flow may trigger one of several actions:

- Send a message
- Update the member profile
- Move them into a new state/context
- Move them into a state of “unknown response”

The last action allows for manual intervention such as calling the member, unsubscribing the member, updating their profile or moving the member through the dialogue to the desired action (e.g. refilling their prescription). This manual fallback allows for both quality control as well as improving the user experience for those members that have difficulty using the mobile interface. The following table lists a few samples of rules used within the Engagement Engine:

**Rules**	**Regular Expression**
Side Effect	`^[\s\S]*(side effect|bloat|nausea|dizzy|light headed|fuzzy|fuzziness|pain|sick|worse)[\s\S]*$`
Forgot	`^[\s\S]*(forget|forgot|reminder|blank)[\s\S]*$`
Cost	`^[\s\S]*(afford|cost|money|aford|monie|pay|expensive)[\s\S]*$`

Over one-third of the dialogues resulted in responses (37.4%) from the patients. The following table shows the breakdown of the final state as recorded within the Engagement Engine. As patients responded, they were moved from state to state based on their response. Pharmacy staff followed up with these patients and prioritized them based on internal guidelines.

**Final State**	**Percentage**
Refill	18.1%*
Change Refill	3.4%
Barrier to Adherence: Cost	0.1%
Barrier to Adherence: Forgot	0.1%
DOB Matched - Didn't Complete	6.9%
DOB No Match	5.2%
Other response	3.7%

No response	62.6%

* There is a difference between "refill requests" via the text solution (18.1%) and "completed refills" that were processed by the pharmacy staff and were slightly lower

In addition to measuring response sentiment, we used natural language processing (NLP) to uncover whether there was any uncertainty or confusion about how to move through the refill dialogue. These NLP driven insights helped us to make changes to the messaging and to cut down the number of free text responses that reflected a lack of understanding. For example, in month 1 (December), many members responded that they didn't want to refill before knowing what they would be refilling. In response to their concerns, we changed the message in Month 2 to make it clear that they would be able to view their order and then refill. But members still weren't sure if the acts of viewing and refilling were simultaneous and wanted to be sure they could view *before* refilling. After we made another change in Month 3, there was less confusion.

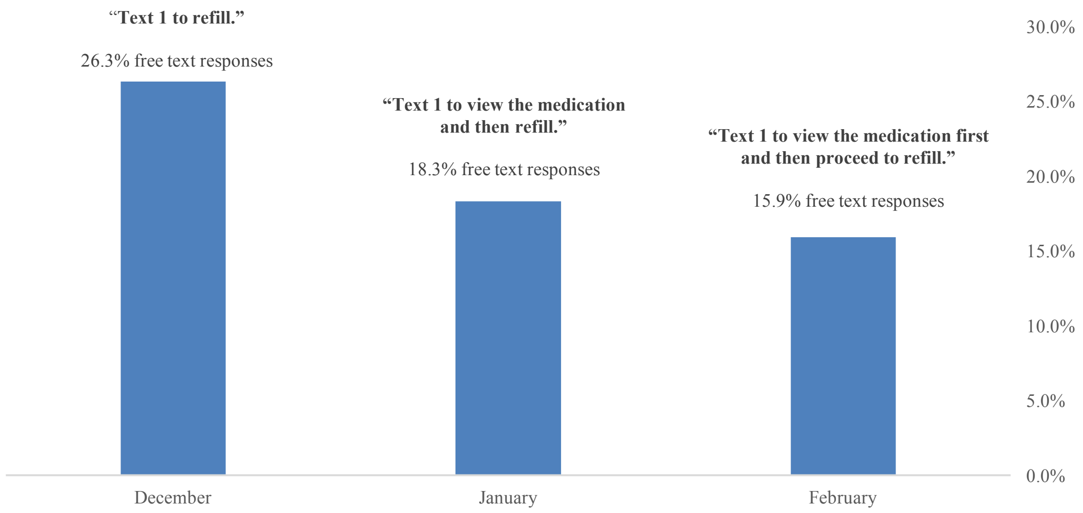

The Refill Automated Dialogue was configured with the following messages shown below and also viewed in the Engagement Console as displayed in the following figures.

REFILL WORKFLOW – PART 1

Target Audience
- **LIST 1 (New Start)**
- **LIST 2 (Near Goal)**
- **LIST 3 (Non-Adherent)**

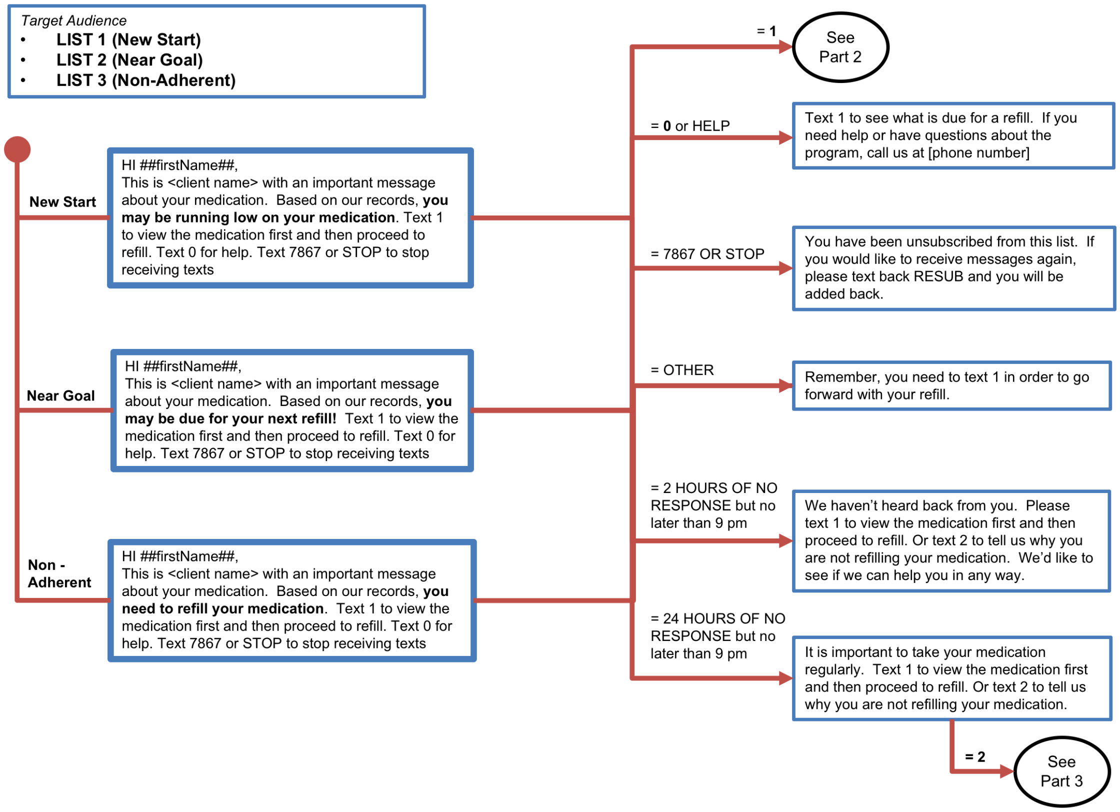

REFILL WORKFLOW – PART 2

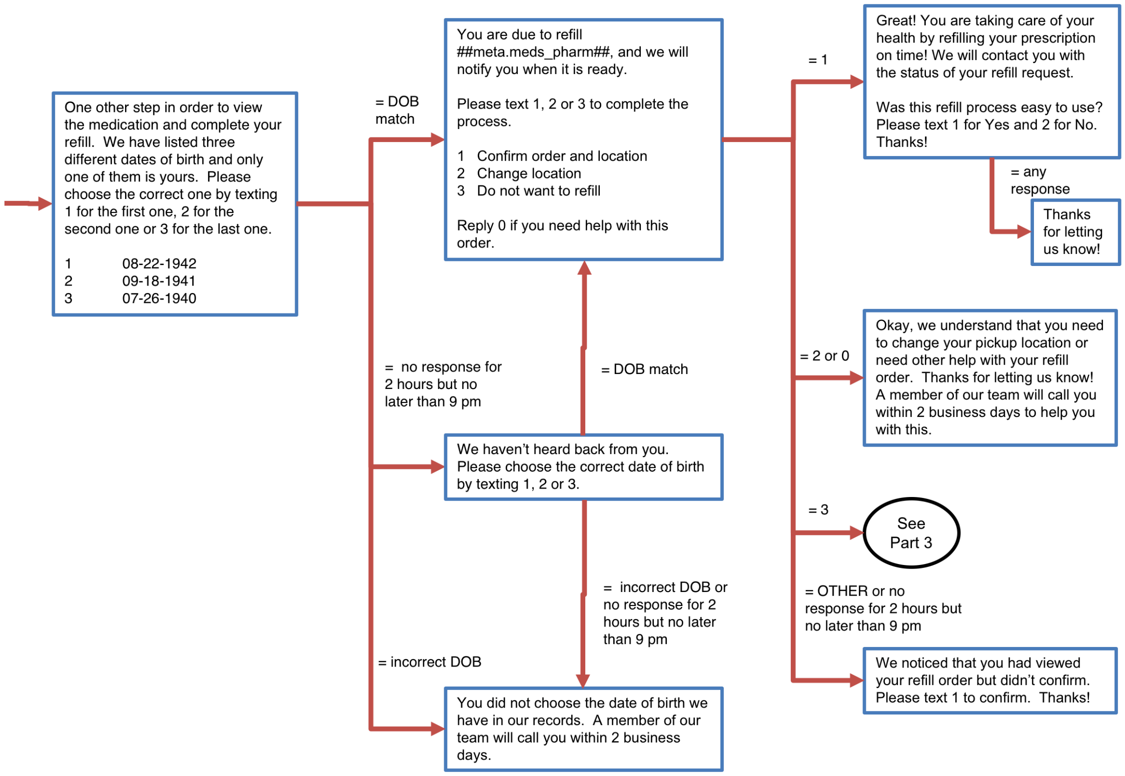

REFILL WORKFLOW – PART 3

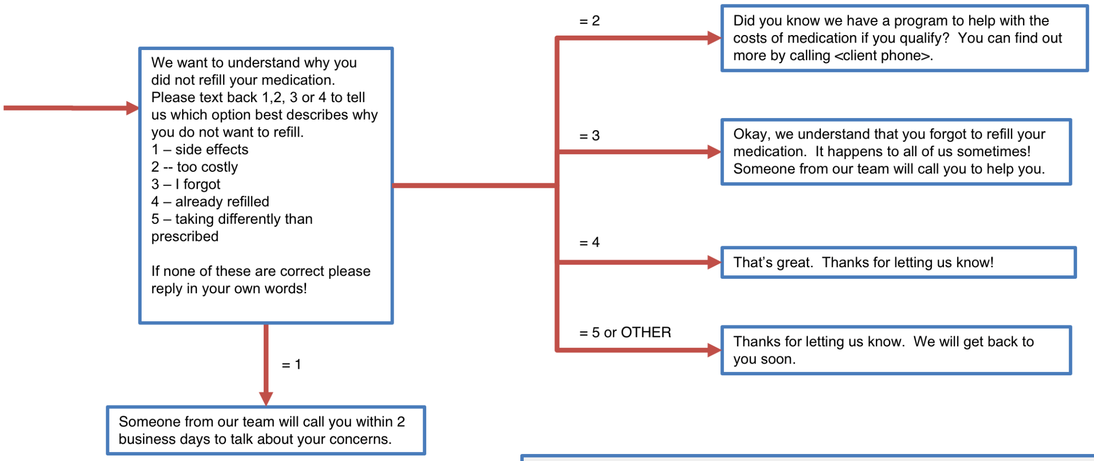

7 Buckets for KP Pharmacy Staff follow-up (searchable in Engagement Console):

1. **Refill** (correct DOB + completed process)
2. **Change** (location or help with order)
3. **DOB** (incorrect DOB)
4. **Incomplete** (correct DOB but didn't complete)
5. **Side Effects** (identified barrier)
6. **Forgot** (identified barrier)
7. **Help**

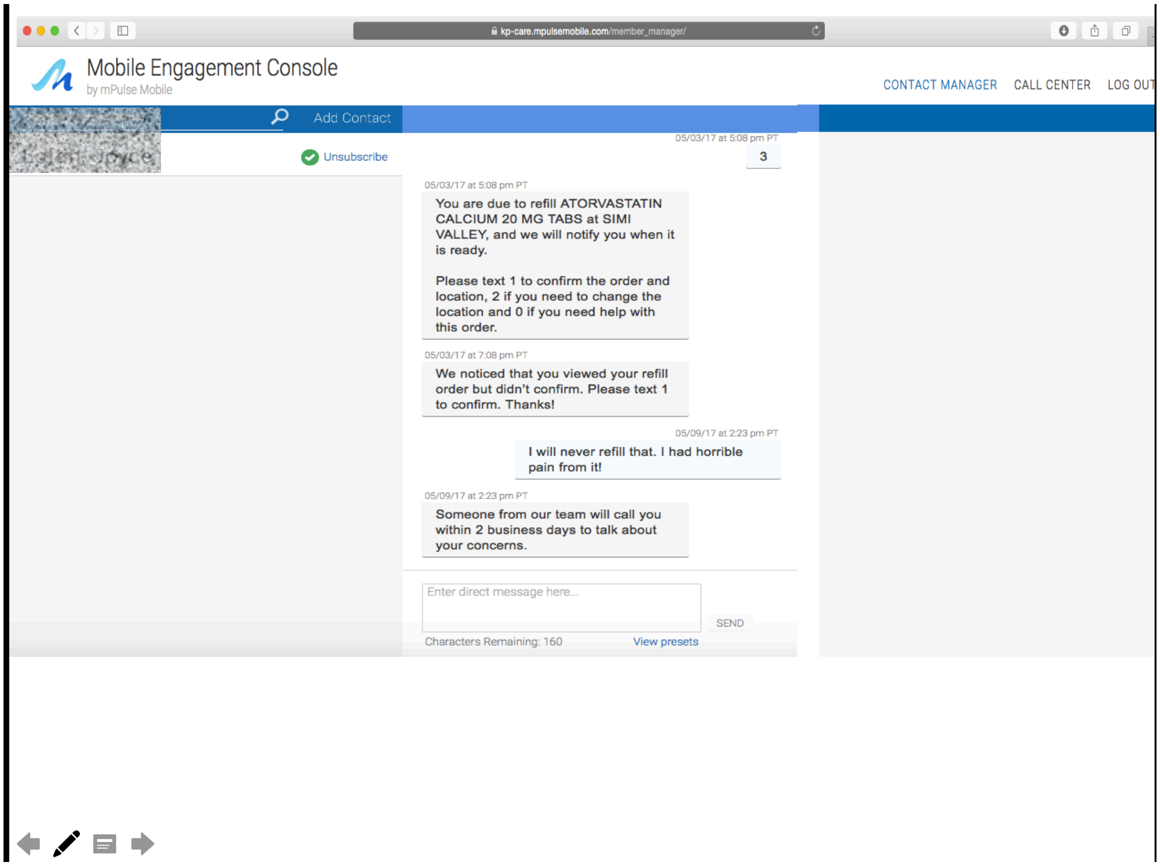
kp-care.mpulsemobile.com/member_manager/
Mobile Engagement Console
by mPulse Mobile
CONTACT MANAGER
CALL CENTER
LOG OUT
Add Contact
Unsubscribe
05/03/17 at 5:08 pm PT
3
05/03/17 at 5:08 pm PT
You are due to refill ATORVASTATIN CALCIUM 20 MG TABS at SIMI VALLEY, and we will notify you when it is ready.
Please text 1 to confirm the order and location, 2 if you need to change the location and 0 if you need help with this order.
05/03/17 at 7:08 pm PT
We noticed that you viewed your refill order but didn't confirm. Please text 1 to confirm. Thanks!
05/09/17 at 2:23 pm PT
I will never refill that. I had horrible pain from it!
05/09/17 at 2:23 pm PT
Someone from our team will call you within 2 business days to talk about your concerns.
Enter direct message here...
SEND
Characters Remaining: 160
View presets

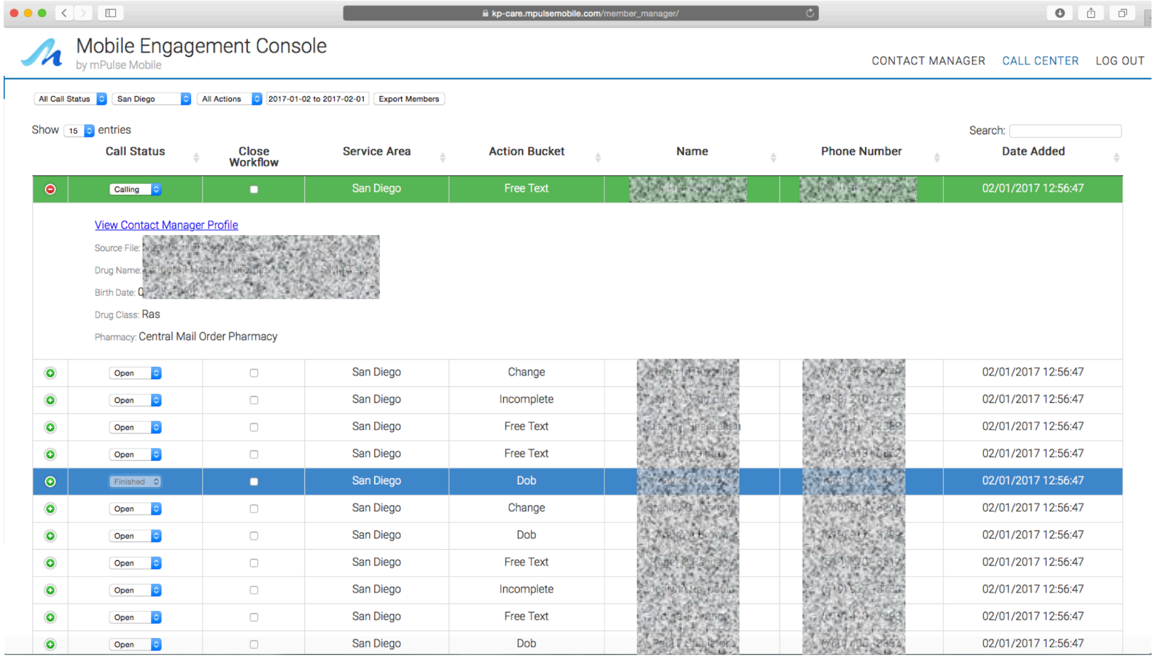
kp-care.mpulsemobile.com/member_manager/
Mobile Engagement Console
by mPulse Mobile
CONTACT MANAGER
CALL CENTER
LOG OUT
All Call Status
San Diego
All Actions
2017-01-02 to 2017-02-01
Export Members
Show 15 entries
Search:
Call Status
Close Workflow
Service Area
Action Bucket
Name
Phone Number
Date Added
Calling
San Diego
Free Text
02/01/2017 12:56:47
View Contact Manager Profile
Source File:
Drug Name:
Birth Date:
Drug Class: Ras
Pharmacy: Central Mail Order Pharmacy
Open | San Diego | Change | 02/01/2017 12:56:47
Open | San Diego | Incomplete | 02/01/2017 12:56:47
Open | San Diego | Free Text | 02/01/2017 12:56:47
Open | San Diego | Free Text | 02/01/2017 12:56:47
Finished | San Diego | Dob | 02/01/2017 12:56:47
Open | San Diego | Change | 02/01/2017 12:56:47
Open | San Diego | Dob | 02/01/2017 12:56:47
Open | San Diego | Free Text | 02/01/2017 12:56:47
Open | San Diego | Incomplete | 02/01/2017 12:56:47
Open | San Diego | Free Text | 02/01/2017 12:56:47
Open | San Diego | Dob | 02/01/2017 12:56:47
